# Genome-Wide Identification and Analysis of the NF-Y Transcription Factor Family Reveal Its Potential Roles in Salt Stress in Alfalfa (*Medicago sativa* L.)

**DOI:** 10.3390/ijms23126426

**Published:** 2022-06-08

**Authors:** Yixin An, Xin Suo, Qichen Niu, Shuxia Yin, Lin Chen

**Affiliations:** 1 School of Grassland Science, Beijing Forestry University, Beijing 100083, China; anyixin@bjfu.edu.cn (Y.A.); suoxin0101@bjfu.edu.cn (X.S.); niuqichen@bjfu.edu.cn (Q.N.); 2 Institute of Animal Science, Chinese Academy of Agricultural Sciences, Beijing 100193, China

**Keywords:** alfalfa (*Medicago sativa* L.), *NF-Y* gene family, abiotic stress, salt tolerance

## Abstract

Nuclear factor Y (NF-Y) is a heterotrimeric transcription factor that plays an important role in various biological processes in plants, such as flowering regulation, drought resistance, and salt stress. However, few in-depth studies investigated the alfalfa *NF-Y* gene family. In this study, in total, 60 *MsNF-Y* genes, including 9 *MsNF-YAs*, 26 *MsNF-YBs*, and 25 *MsNF-YCs,* were identified in the alfalfa genome. The genomic locations, gene structures, protein molecular weights, conserved domains, phylogenetic relationships, and gene expression patterns in different tissues and under different stresses (cold stress, drought stress, and salt stress) of these *NF-Y* genes were analyzed. The illustration of the conserved domains and specific domains of the different subfamilies of the *MsNF-Y* genes implicates the conservation and diversity of their functions in alfalfa growth, development, and stress resistance. The gene expression analysis showed that 48 *MsNF-Y* genes (7 *MsNF-YAs*, 22 *MsNF-YBs*, and 19 *MsNF-YCs*) were expressed in all tissues at different expression levels, indicating that these genes have tissue expression specificity and different biological functions. In total, seven, seven, six, and eight *MsNF-Y* genes responded to cold stress, the ABA treatment, drought stress, and salt stress in alfalfa, respectively. According to the WGCNA, molecular regulatory networks related to salt stress were constructed for *MsNF-YB2*, *MsNF-YB5*, *MsNF-YB7*, *MsNF-YB15*, *MsNF-YC5*, and *MsNF-YC6*. This study could provide valuable information for further elucidating the biological functions of *MsNF-Ys* and improving salt tolerance and other abiotic stress resistance in alfalfa.

## 1. Introduction

Alfalfa (*Medicago sativa* L.) is one of the top-quality forages for livestock, such as dairy cattle, because of its high nutrient content and crude protein content and has been hailed as the ‘king of forages’ [1,2]. However, alfalfa is frequently affected by various abiotic stresses, such as drought, cold, and salt, during alfalfa growth and development, which seriously affect the yield and quality of alfalfa [3]. Alfalfa has formed complex gene regulatory networks in response to increasingly deteriorating growth environments over a long period of evolution [3]. In addition, with the reporting of the alfalfa reference genome, several transcription factor families, such as WRKY, CBF, SPL, and MYB, have been identified to be involved in abiotic stress processes in alfalfa [3]. However, our knowledge of the complex transcriptional regulatory network is limited.

The nuclear factor Y (NF-Y) transcription factor, also known as the CCAAT-binding factor (CBF) or the heme activator protein (HAP), is widespread in eukaryotes. NF-Y consists of three distinct subunits, NF-YA, NF-YB, and NF-YC [4]. The subfamily (NF-YA, NF-YB, and NF-YC) members can be identified by the sequence length and conserved motifs or domains. In general, NF-YA subunits have a longer sequence than the NF-YB and NF-YC sequences [5]. The conserved region in the NF-YA subunits usually consists of two conserved domains, α1 and α2, which include 53 amino acids [5]. The NF-YB subunit is shorter in sequence length, and the conserved protein domains in the NF-YB and NF-YC subunits are extremely similar to the H2B and H2A histones, respectively [5]. NF-YB and NF-YC first form dimers in the cytoplasm and then they can be located into the nucleus and interact with NF-YA to form a heterotrimeric complex [5]. This typical NF-Y heterotrimeric complex activates or represses the expression of downstream genes by binding CCAAT sequences located in the promoters of target genes [5,6]. In addition, different NF-Y subunits can form complexes with other transcription factors, thereby regulating the expression of downstream genes [7,8].

The NF-Y transcription factor has been confirmed to be involved in multiple bio-logical processes, such as plant seed development, flowering, and fruit development [9,10,11,12]. *AtNF-YB9*, the first NF-Y gene to be cloned in plants, is involved as a key regulatory transcription factor in *Arabidopsis* seed development [13,14]. The mutation of *OsNF-YB9*, which is the homologous gene of *AtNF-YB9* in rice, could cause abnormal seed development, such as seeds becoming longer, narrower, and thinner and exhibiting a higher chalkiness ratio [11]. *AtNF-YB6* has also been found as a regulator of embryo development and specifically expressed in developing *Arabidopsis* embryos [15]. *FtNF-YB7*, which is the homologous gene of *AtNF-YB9* in buckwheat, was also specifically expressed in fruit and indicated its important role in fruit development [16]. In recent years, the role of NF-Y gene family members during abiotic stress in plants has received much attention. *AtNF-YA5* was highly expressed in vascular tissues and guard cells and was induced by drought stress [17]. The overexpression of *AtNF-YA5* could reduce leaf water loss and increase the drought tolerance [17]. Overexpression of *GmNF-YA5*, which is the homologous gene of AtNF-YA5 in soybean, in *Arabidopsis* and soybean could enhance the drought tolerance in seedlings by decreasing the water loss from leaves [18]. *AtNF-YB1* in *Arabidopsis*, *ZmNF-YB2* in maize, and *GmNF-YB01* in soybean are homologous and play an important role in drought stress [10,19]. The overexpression of *AtNF-YB2* and *AtNF-YB3* in *Arabidopsis* increased drought and heat tolerance, respectively [20]. The overexpression of the *ZmNF-YB16* genes in maize improved drought resistance and yield [21]. GmNF-YC14, a member of the soybean NF-YC subunit family, was found to be significantly upregulated simultaneously in response to drought and salt stress and ABA treatment [22]. GmNF-YC14 was able to interact with GmNF-YB2 and GmNF-YA16 in soybean to form an intact and active NF-Y transcriptional complex [22]. This NF-Y complex could regulate the ABA signaling pathway mediated by ABA receptor PYR/PYL under drought stress to enhance drought and salt tolerance in soybean [22]. With the rapid development of genomics, an increasing number of NF-Y gene families have been identified and analyzed at the genomic level; for example, 38 NF-Y gene family members were identified in the bitter buckwheat genome [16], 34 NF-Y genes were identified in the grape genome [23], and 25 genes were identified in the castor bean genome, and their functions have been preliminarily analyzed [24]. However, the basic information and functions of NF-Y transcription factors in alfalfa remain poorly understood.

In this study, the role of *MsNF-Ys* in the regulation of multiple abiotic stresses of plants, especially salt stress in alfalfa, was investigated. In total, 60 *MsNF-Y* genes (9 *MsNF-YAs*, 26 *MsNF-YBs*, and 25 *MsNF-YCs*) were identified in the alfalfa reference genome based on bioinformatics methods. The gene structure, motif composition, conserved domains, and *cis*-acting elements in the promoter regions of the different NF-Y subfamilies in alfalfa were analyzed. In addition, a homology analysis and phylogenetic tree analysis of *NF-Y* genes from alfalfa, *Arabidopsis*, buckwheat, rice, and soybean were conducted to predict the function of *MsNF-Ys*. *NF-Y* gene members were identified to be involved in different biological processes by an expression pattern analysis in different tissues of alfalfa under different treatments (cold, ABA, drought, and salt). More importantly, the expression of *MsNF-Y* genes during salt stress was comprehensively analyzed by a WGCNA, indicating that some *MsNF-Y* genes are involved in the alfalfa salt stress process. This study may provide valuable information for the identification of candidate *NF-Y* genes involved in the regulation of abiotic stress (especially salt stress) in alfalfa.

## 2. Results

### 2.1. Identification of MsNF-Y Family Genes

In total, 60 *MsNF-Y* genes, including 9 *MsNF-YAs*, 26 *MsNF-YBs*, and 25 *MsNF-YCs*, were identified from the alfalfa reference genome (Xinjiangdaye). Then, we named these 60 *MsNF-Y* genes (the NF-YA subfamily was named *MsNF-YA1* to *MsNF-YA9*, the NF-YB subfamily was named *MsNF-YB1* to *MsNF-YB26*, and the NF-YC subfamily was named *MsNF-YC1* to *MsNF-YC25*) according to their chromosomal locations and subfamilies (Table 1).

The protein lengths of the 60 *MsNF-Y* genes showed a wide distribution, ranging from 136 AA (MsNF-YB5) to 400 AA (MsNF-YC14) (Table 1 and Table S1). The protein length of the MsNF-YA members ranged from 312 AA to 334 AA, while the protein length of the MsNF-YB members ranged from 136 AA to 237 AA, and the MsNF-YC members were distributed from 160 AA to 400 AA. Among the three MsNF-Y subfamilies, the MsNF-YA subfamily member proteins had the longest average length of 328 AA, whereas MsNF-YB had the shortest protein with an average length of 182.3 AA, and MsNF-YC had an intermediate average length of 240.9 AA. The predicted protein molecular weight (MW) results showed that the MWs of the 60 MsNF-Ys were widely distributed from 15.28 kDa to 43.94 kDa, with the MsNF-YA subfamily distribution ranging from 36.08 kDa to 36.31 kDa and a mean of 35.62 kDa, the MsNF-YB subfamily distribution ranging from 15.28 kDa to 26.28 kDa and a mean of 19.57 kDa, and the MsNF-YC subfamily distribution ranging from 17.32 kDa to 43.94 kDa and a mean of 30.81 kDa. The predicted pI of the 60 MsNF-Ys in alfalfa ranged from 4.61 (MsNF-YB5) to 8.48 (MsNF-YA9) (Table 1).

### 2.2. Analysis of the Motifs and Conserved Domains of MsNF-Y Family Members

**Figure 1:**
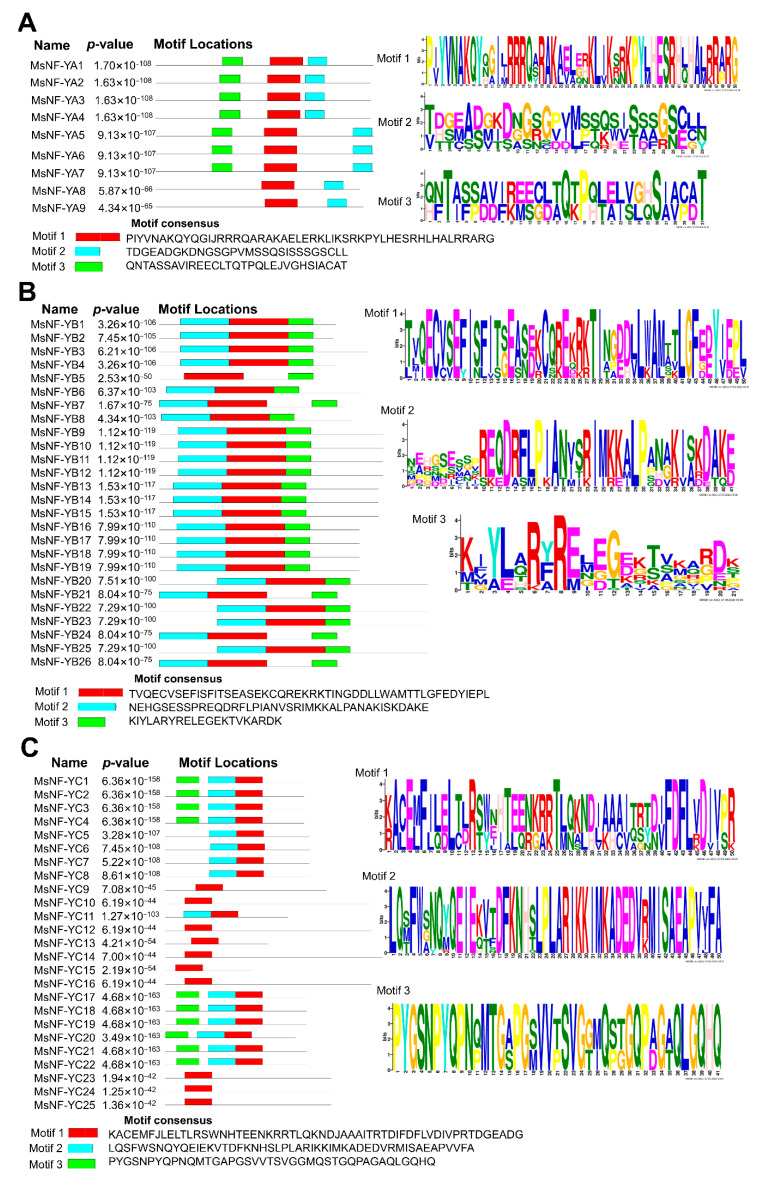
Motif analysis of MsNF-Y proteins. (**A**) MsNF-YA proteins; (**B**) MsNF-YB proteins; (**C**) MsNF-YC proteins.

To analyze the motif composition of these MsNF-Y proteins, MEME software was used to identify the motifs. The results showed that different subfamilies had different motif compositions (Figure 1). In the MsNF-YA group, motif 1 and motif 2 were found in all members (Figure 1A). MsNF-YA8 and MsNF-YA9 did not have motif 3. In the MsNF-YB subfamily, all members, except for MsNF-YB5, contained the three motifs identified (Figure 1B). In the MsNF-YC subfamily, the composition of the motifs in these proteins was relatively diverse. There were only 10 MsNF-YC proteins with one motif and five MsNF-YC proteins with two motifs (Figure 1C).

**Figure 2:**
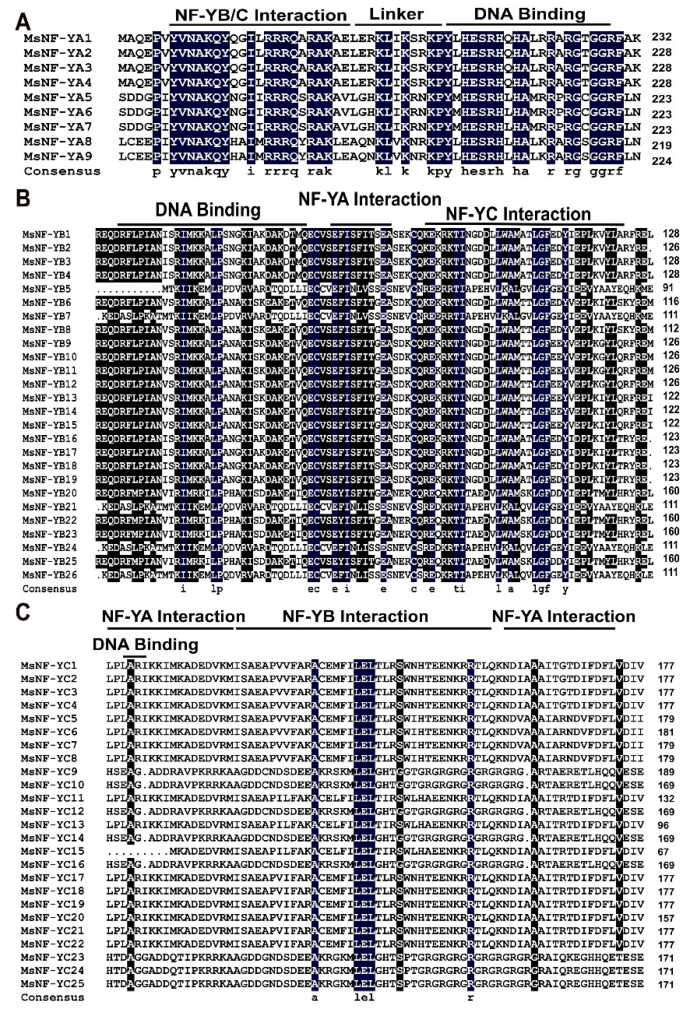
Conserved domain alignments of MsNF-Y members. (**A**) MsNF-YA proteins; (**B**) MsNF-YB proteins; (**C**) MsNF-YC proteins.

To analyze the conserved domains in the different NF-Y subfamilies, multiple alignments were conducted by using DNAMAN software. The results showed that there were a few conserved domains in the MsNF-Y proteins, indicating that these proteins belong to the same subgroups (Figure 2). For example, the MsNF-YA proteins have conserved domains, including one domain for the protein–protein interaction of NF-YB/C and another for the DNA-binding domain (Figure 2A). The MsNF-YB proteins have one domain for DNA binding and another domain for interacting with NF-YA and NF-YC proteins (Figure 2B). Among the MsNF-YC proteins, the results are the same (one domain for DNA binding and another domain for the interaction of NF-YA/B) but with lower conservation compared with that in the MsNF-YA and MsNF-YB subfamilies, implying a probably more abundant functional role in alfalfa (Figure 2C).

### 2.3. Phylogenetic Relationships and Gene Structure of the MsNF-Y Family Members

**Figure 3:**
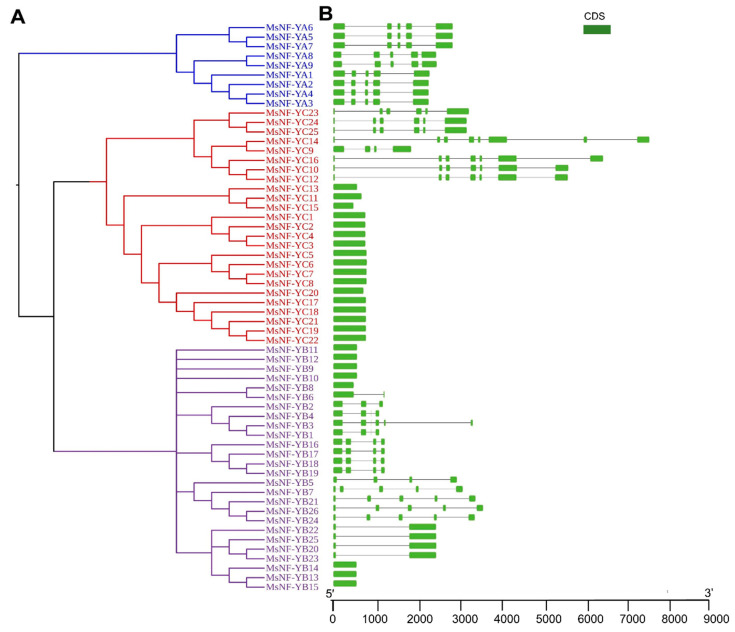
Phylogenetic analysis and gene structure of *MsNF-Y* genes. (**A**) Phylogenetic tree of MsNF-Ys; (**B**) Gene structure of alfalfa *NF-Y* genes. The green box indicates exons, and the black lines indicate introns.

To understand the phylogenetic relationships among the MsNF-Ys, phylogenetic trees were constructed by using MEGA 5.0 software based on the protein sequences. The results showed that the neighbor-joining tree was divided into three branches corresponding to three different subfamilies (MsNF-YAs, MsNF-YBs, and MsNF-YCs) (Figure 3A). In the MsNF-YA subfamily, there are three major groups. One group contains MsNF-YA6, MsNF-YA5, and MsNF-YA7, another group contains MsNF-YA8 and MsNF-YA9, and the third group contains MsNF-YA1, MsNF-YA2, MsNF-YA3, and MsNF-YA4. In the MsNF-YB subfamily, we found that MsNF-YB11, MsNF-YB12, MsNF-YB9, and MsNF-YB10 were relatively independent of the five groups formed by the other MsNF-YBs. In the MsNF-YC subfamily, there are two major groups. The first group contains MsNF-YC23, MsNF-YC24, MsNF-YC25, MsNF-YC14, MsNF-YC9, MsNF-YC16, MsNF-YC10, and MsNF-YC12. The other MsNF-YCs formed the second group.

We used TBtools to analyze the gene structures of the *MsNF-Ys*. The results showed that all *MsNF-YAs* contained five exons and four introns (Figure 3B). Eight *MsNF-YB* genes contained only one exon, and five *MsNF-YB* genes contained two exons and one intron. The other *MsNF-YBs* contained three or more exons, and *MsNF-YB3* harbored six exons and five introns (Figure 3B). Among the 25 *MsNF-YC* genes, 17 members contained only one exon, while *MsNF-YC14*, *MsNF-YC16* (also including *MsNF-YC10* and *MsNF-YC12*), *MsNF-YC23* (also including *MsNF-YC24* and *MsNF-YC25*), and *MsNF-YC9* contained eight, seven, six, and four exons, respectively (Figure 3B). Based on the phylogenetic tree and gene structure, we found that the same tree groups had the same gene structure (Figure 3).

### 2.4. Genome Distribution and Gene Duplication Events of the MsNF-Ys in the Alfalfa Genome

**Figure 4:**
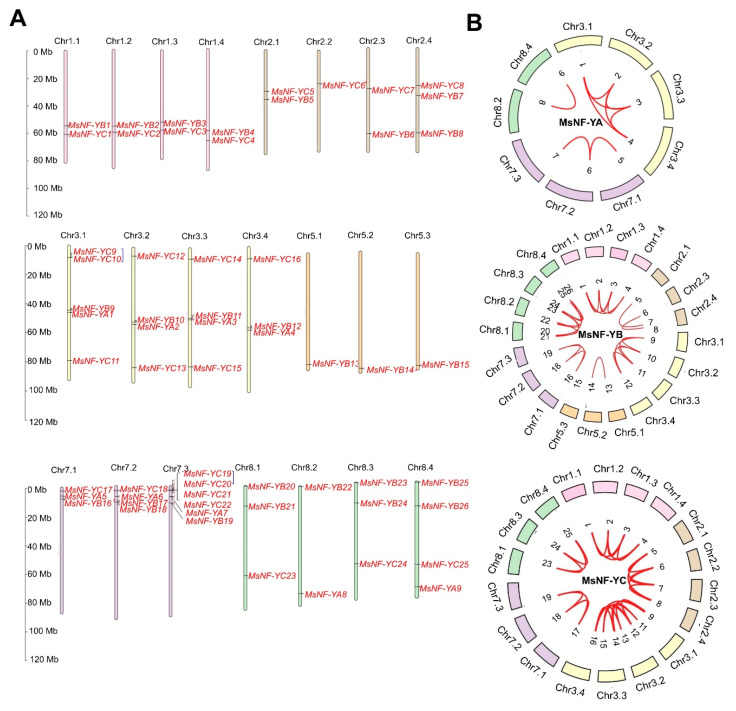
Genome locations and duplication analysis of 60 *MsNF-Y* genes in the alfalfa genome. (**A**) The physical locations of the 60 *MsNF-Y* genes. The blue lines connect the corresponding two pairs of paralogous genes in the same chromosome. (**B**) Duplication analysis of *MsNF-Y* genes on different chromosomes. From left to right are the NF-YA, NF-YB, and NF-YC subfamilies.

To show the genome distribution of the 60 *MsNF-Y* genes in the alfalfa genome, the TBtools program was used. The results showed that the 60 *MsNF-Y* genes were distributed unevenly on the 32 alfalfa chromosomes, except for chr4.1, chr4.2, chr4.3, chr4.4, chr5.4, chr6.1, chr6.2, chr6.3, chr6.4, and chr7.4 (Figure 4A). Six *NF-Y* genes were on chr7.3, while on the other chromosomes there were no more than five *NF-Y* genes.

In total, 60 duplication events were found among these *MsNF-Y* genes in the alfalfa genome (Figure 4). Two duplication events (*MsNF-YC9* and *MsNF-YC10* on chr3.1 and *MsNF-YC19* and *MsNF-YC20* on chr7.3) were formed in the *NF-YC* genes on the same chromosome, and 58 duplication events were formed by different *NF-Y* genes on different chromosomes (Figure 4B and Table S2). Among the 58 duplication events, 9 duplication events were formed by the 9 *MsNF-YA* genes, 25 duplication events were formed by the 24 *MsNF-YB* genes, and 24 duplication events were formed by the 21 *MsNF-YC* genes. To understand the evolution type among these duplication events on different chromosomes, the Ka, Ks, and Ka/Ks values were calculated in this study. The results showed that among the 58 duplication events, 41 duplication events were estimated as purifying selection, 11 events were neutral evolution, and 6 events were positive selection (Table S2).

### 2.5. cis-Element Analysis in the Promoter Regions of MsNF-Y Genes

**Figure 5:**
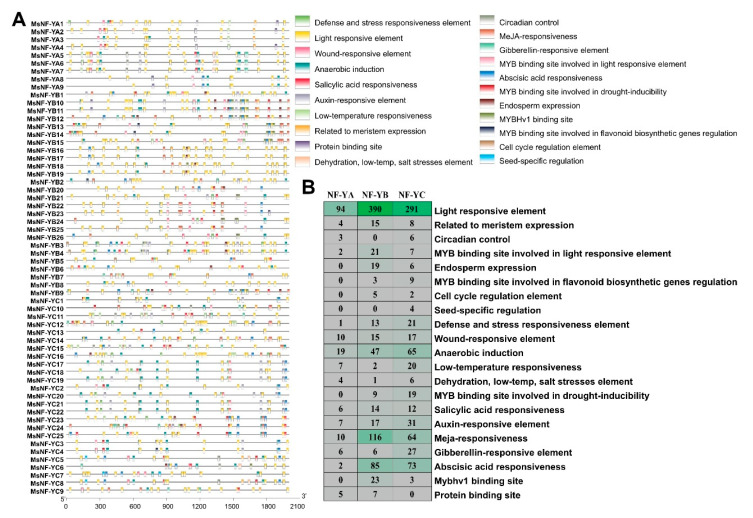
*cis*-element analysis of the 60 *MsNF-Ys* promoter regions. (**A**) The locations of these *cis* elements in the *MsNF-Y* promoter regions; the boxes with different colors indicate different *cis* elements. (**B**) Statistical analysis of different types of *cis* elements.

To clarify the transcriptional regulation of the *MsNF-Y* genes, the *cis* elements in the promoter regions were identified by using the PlantCare program. In total, 1679 *cis* elements belonging to 21 different categories were found in the promoter regions of the 60 *MsNF-Y* genes (Figure 5A). Light-responsive elements existed in the promoter regions of all *MsNF-Ys,* and there were 94, 390, and 291 light-responsive elements in *MsNF-YA*, *MsNF-YB*, and *MsNF-YC*, respectively (Figure 5B). In addition to light-responsive elements, MeJA-responsive elements are the second largest category in the promoter regions of *MsNF-Ys*. In total, 38 *MsNF-Y* genes (5 *MsNF-YAs*, 22 *MsNF-YBs*, and 11 *MsNF-YCs*) contained MeJA-responsive elements in the promoter regions (Figure 5B). In total, 160 abscisic acid response elements (ABREs) were found in the promoter regions of 51 *MsNF-Y* genes (2 *MsNF-YAs*, 25 *MsNF-YBs*, and 24 *MsNF-YCs*). There were 55 auxin-responsive elements and 39 gibberellin-responsive elements in the promoter regions of 29 and 27 *MsNF-Y* genes, respectively (Figure 5A). The promoter regions of 24, 11 and 21 *MsNF-Y* genes harbored MYB transcription-factor-binding sites related to drought inducibility, flavonoid biosynthetic gene regulation, and light-responsive elements, respectively. Many stress response elements were also found in the promoter regions of the *MsNF-Ys* (Figure 5B). The promoter regions of 19, 22, and 10 *MsNF-Y* genes contained 29 low temperature response elements (LTRs), 35 defense and stress responsiveness elements (TC-rich repeats), and 11 dehydration, low temperature, and salt stress elements (DREs), respectively. These results indicate that *MsNF-Ys* not only participate in the growth and development of alfalfa but also play a key role in various stresses.

### 2.6. NF-Y Gene Evolutionary Analysis of Different Plant Species

**Figure 6:**
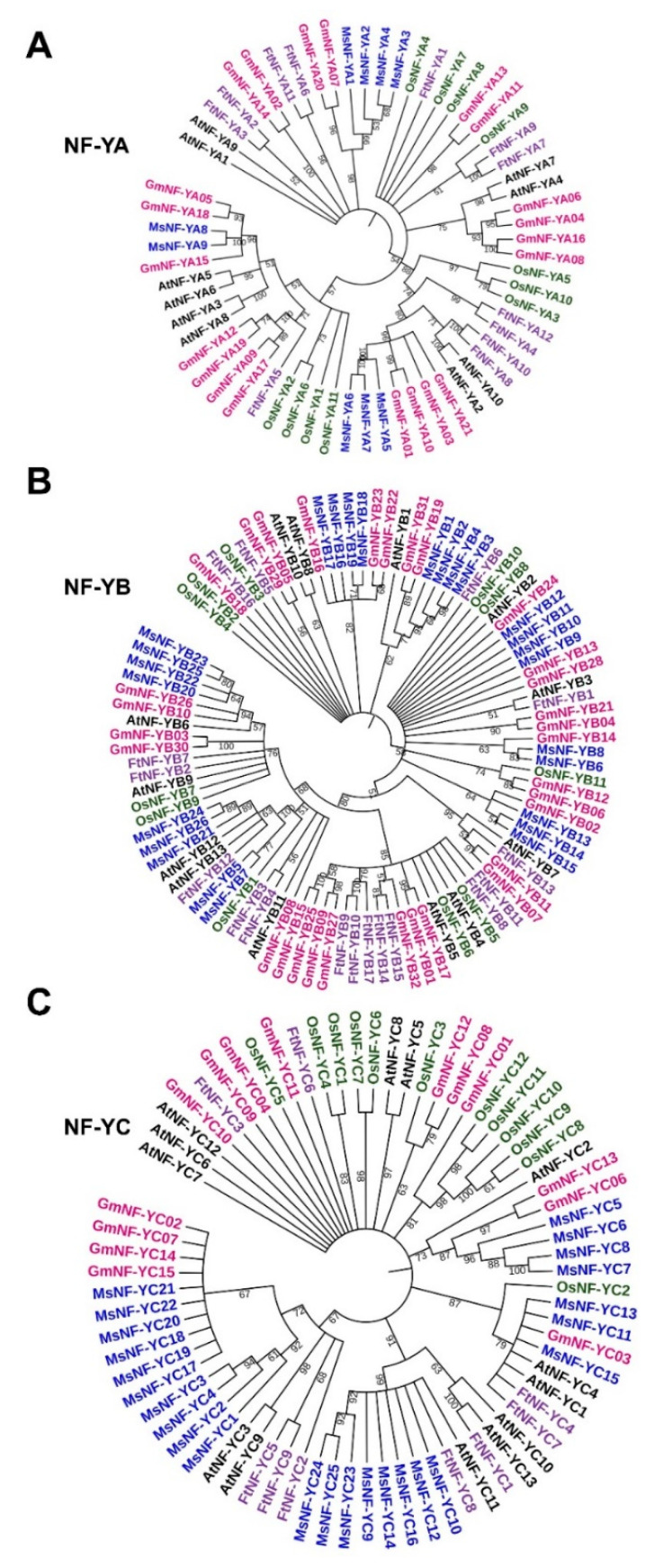
Phylogenetic relationship of NF-Y proteins from alfalfa, *Arabidopsis*, rice, soybean, and Tartary buckwheat. (**A**) NF-YA subfamily. (**B**) NF-YB subfamily. (**C**) NF-YC subfamily.

To understand the evolutionary relationships of the *NF-Y* gene in different plant species and predict the function of *MsNF-Ys*, the NF-Y protein sequences from rice, soybean, *Arabidopsis*, Tartary buckwheat, and alfalfa were collected (Table S1). Based on the phylogenetic analysis, the results showed that most MsNF-Y members were close to the homolog GmNF-Ys in the soybean genome (Figure 6). In the NF-YA subfamily, MsNF-YA1/2/3/4 were closer to GmNF-YA07/20, MsNF-YA8/9 were closer to GmNF-YA05/18, and MsNF-YA5/7/6 were closer to GmNF-YA01/10/03/21 (Figure 6A). In the NF-YB subfamily, MsNF-YB16/17/18/19 were closer to GmNF-YB16, MsNF-YB6/8 were closer to GmNF-YB14, and MsNF-YB20/22/23/25 were closer to GmNF-YB10/26 (Figure 6B). In the NF-YC subfamily, MsNF-YC5/6/7/8 were closer to GmNF-YC06/13. These results suggest that these alfalfa *MsNF-Y* genes may perform biological functions similar to those in soybean (Figure 6). In addition, some *MsNF-Y* genes were closer to homologs in other plant species. For example, MsNF-YB21/24/26 were closer to FtNF-YB12 from Tartary buckwheat and AtNF-YB12/13 from *Arabidopsis*. These results provide valuable information for further functional analysis of these *MsNF-Ys*.

### 2.7. Expression Analysis of MsNF-Y Genes in Different Tissues from Alfalfa

**Figure 7:**
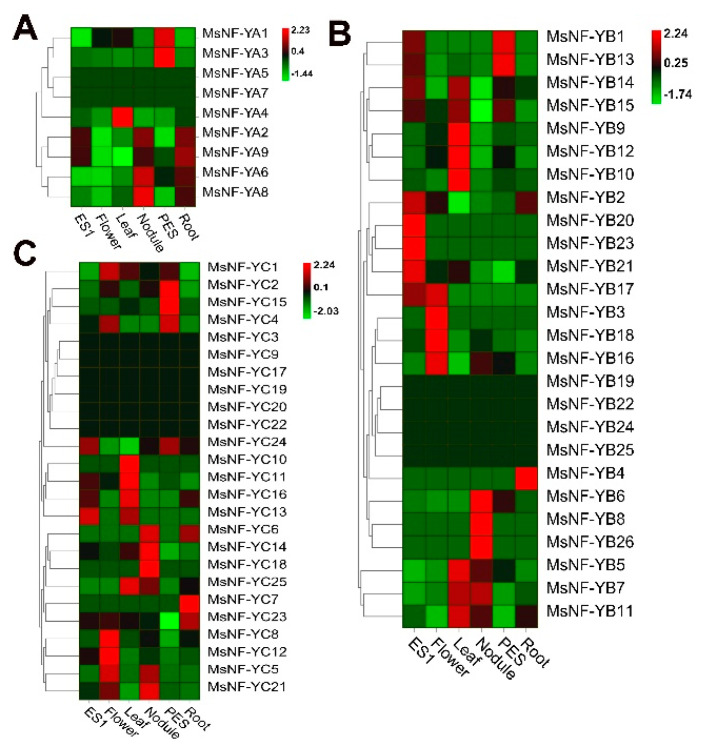
Expression pattern analysis of the 60 *MsNF-Y* genes in various alfalfa tissues (ES1, elongated stem; flower; leaf; nodule; PES, preelongated stem; and root). (**A**) *MsNF-YA* genes. (**B**) *MsNF-YB* genes. (**C**) *MsNF-YC* genes.

To investigate the expression pattern of these *MsNF-Y* genes, the global gene expression in different tissues (root, flower, nodule, leaf, ES1, and PES) of alfalfa was collected and analyzed. The results showed that the 60 *MsNF-Y* genes had significantly different expression patterns and indicated that these genes played different roles in alfalfa development (Figure 7). Among the nine *MsNF-YA* genes, two *MsNF-YAs* (*MsNF-YA5* and *MsNF-YA7*) were not expressed in the six tissues in this study. *MsNF-YA1* and *MsNF-YA3* exhibited high expression levels in the PES, and *MsNF-YA4* had a higher expression level in the leaf. *MsNF-YA2/9/6/8* had a similar expression pattern and were mainly expressed in the roots and nodules (Figure 7A). Among the twenty-six *MsNF-YB* genes, four genes (*MsNF-YB19/22/24/25*) were not expressed in these tissues (Figure 7B). *MsNF-YB4* was only expressed in the roots, and *MsNF-YB6/8/26* were only expressed in the nodules. Based on the expression patterns, these *MsNF-YB* genes were clustered into different groups. *MsNF-YB1/MsNF-YB13* were mainly expressed in ES1 and PES, and *MsNF-YB14/15* were mainly expressed in ES1, PES, and leaves. *MsNF-YB9/12/10* were mainly expressed in the leaf, while *MsNF-YB17/3/18/16* were mainly expressed in the flower, and *MsNF-YB6/8/26* were mainly expressed in the nodule (Figure 7B). Among the twenty-five *MsNF-YC* genes, six genes (*MsNF-YC3*, *MsNF-YC9*, *MsNF-YC17*, *MsNF-YC19*, *MsNF-YC20*, *MsNF-YC22*, and *MsNF-YC24*) were not expressed in these tissues (Figure 7C). *MsNF-YC7* was only expressed in the root, while *MsNF-YC10* is only expressed in the leaf. The other *MsNF-YC* genes were expressed in at least two different tissues (Figure 7C). These results indicate that these *MsNF-Y* genes perform different functions in alfalfa growth and development.

### 2.8. Expression Analysis of MsNF-Y Genes in Alfalfa under Different Abiotic Stresses

**Figure 8:**
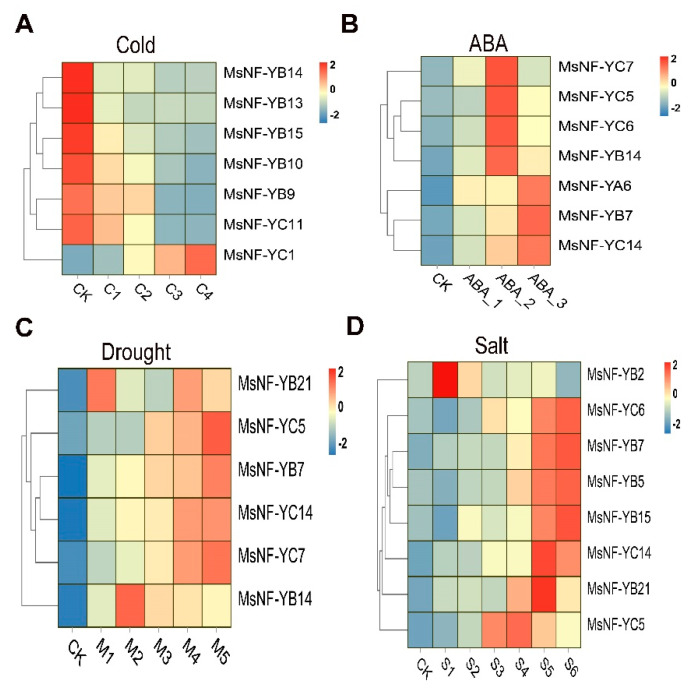
Candidate *MsNF-Y* genes responsible for different abiotic stresses. (**A**) *MsNF-Y* genes involved in the cold stress response. (**B**) *MsNF-Y* genes involved in the ABA treatment response. (**C**) *MsNF-Y* genes involved in the drought response. (**D**) *MsNF-Y* genes involved in the salt stress response.

To clarify the role of the *MsNF-Y* genes under different abiotic stresses, we collected transcriptome data under cold, ABA, drought, and salt stress. The results showed that multiple *MsNF-Y* genes are involved in different abiotic stress processes (Figure 8). The expression levels of seven *MsNF-Y* genes (five *MsNF-YB* genes and two *MsNF-YC* genes) significantly changed under cold stress (Figure 8A). Among the seven genes, the expression levels of six genes decreased as the cold stress treatment time increased, and only one gene (*MsNF-YC1*) showed an increase as the cold stress treatment time increased (Figure 8A). Under the ABA treatment, the expression levels of seven genes (one *MsNF-YA* gene, two *MsNF-YB* genes, and four *MsNF-YC* genes) were significantly upregulated (Figure 8B). The expression levels of six *MsNF-Y* genes (three *MsNF-YB* genes and three *MsNF-YC* genes) were significantly upregulated under drought stress (Figure 8C). *MsNF-YB21* and *MsNF-YB14* expression abundance was significantly upregulated in the early stage of drought stress. The gene expression levels of the other four genes (*MsNF-YC5*, *MsNF-YB7*, *MsNF-YC14*, and *MsNF-YC7*) were significantly upregulated in the later stage of drought stress. Under salt stress, the expression levels of eight *MsNF-Y* genes (five *MsNF-YB* genes and three *MsNF-YC* genes) were significantly upregulated (Figure 8D). *MsNF-YB2* gene expression was significantly upregulated in the early stage of salt stress. *MsNF-YC5* and *MsNF-YB21* gene expression first increased and then decreased as the salt treatment time increased. The expression levels of the other five genes were significantly upregulated in the later stage of salt stress (Figure 8D).

Combining these different abiotic stress results, we also found multiple *MsNF-Y* genes involved in two or more stresses. *MsNF-YB14* responds to both cold and drought stresses, *MsNF-YB15* responds to both cold and salt stresses, and *MsNF-YB7*, *MsNF-YC5*, and *MsNF-YC14* respond to both drought and salt stresses.

### 2.9. Important Candidate MsNF-Y Genes in Salt Stress

**Figure 9:**
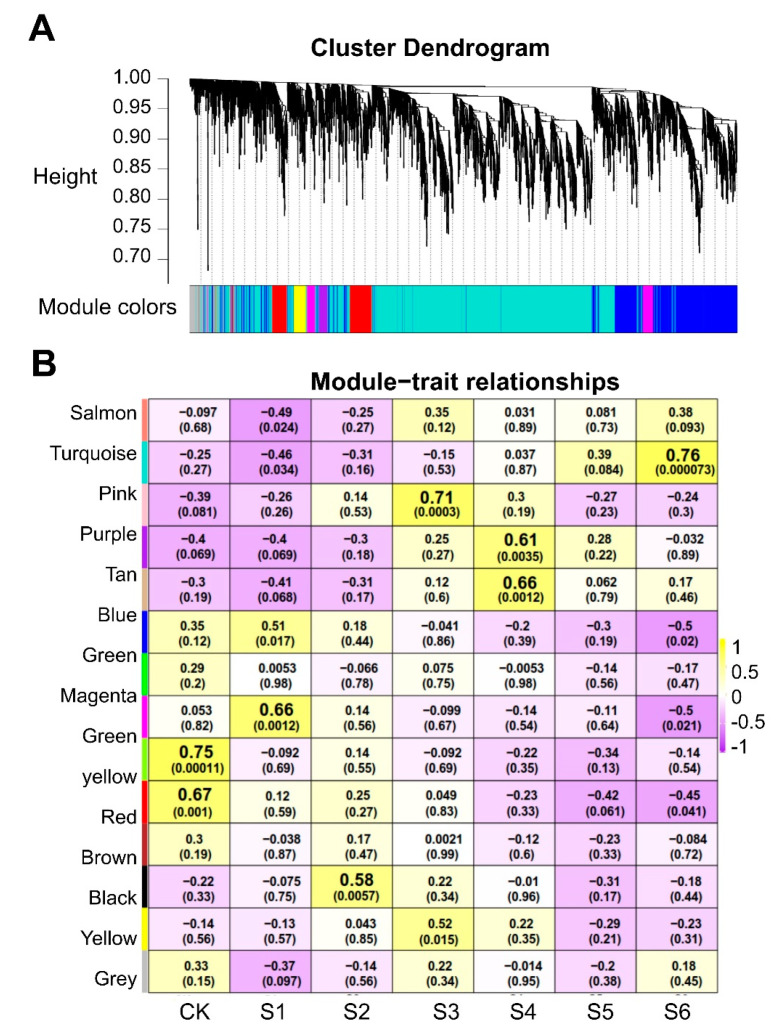
Weighted correlation network analysis (WGCNA) coexpression network and module-trait correlation analysis under salt stress. (**A**) Hierarchical cluster tree showing the coexpression modules. Different colors indicate different modules. (**B**) Correlation analysis between different modules and salt stress duration. The top number in the cell represents the correlation coefficient and the bottom number indicates the *p* value.

To further identify candidate genes among the eight differentially expressed *MsNF-Y* genes in salt stress, a weighted gene coexpression network analysis (WGCNA) was conducted in this study. The results showed that these genes were divided into 14 coexpression modules (Figure 9A). The correlation analysis between the modules and the salt stress treatment time revealed that a few modules were significantly correlated with salt stress (Figure 9B). The magenta and black modules were significantly correlated with the early stage (Tables S1 and S2) of salt stress (R = 0.66, *p* = 1.2 × 10^−3^; R = 0.58, *p* = 5.7 × 10^−^^3^). The pink, purple, and tan modules were significantly correlated with the middle stage (Tables S3 and S4) of salt stress (R = 0.71, *p* = 3.0 × 10^−4^; R = 0.61, *p* = 3.5 × 10^−3^; R = 0.66, *p* = 1.2 × 10^−3^). The turquoise module had a higher correlation with the later stage of salt stress (R = 0.76, *p* = 7.3 × 10^−5^).

Among these significant modules, six *MsNF-Y* genes were included. *MsNF-YB2*, which responds to salt stress at the early stage, was found in the magenta module; *MsNF-YC5*, which responds to salt stress at the middle stage, was found in the purple module; and the other four *MsNF-Y* genes (*MsNF-YB5*, *MsNF-YB7*, *MsNF-YB15*, and *MsNF-YC6*) were located in the turquoise module.

**Figure 10:**
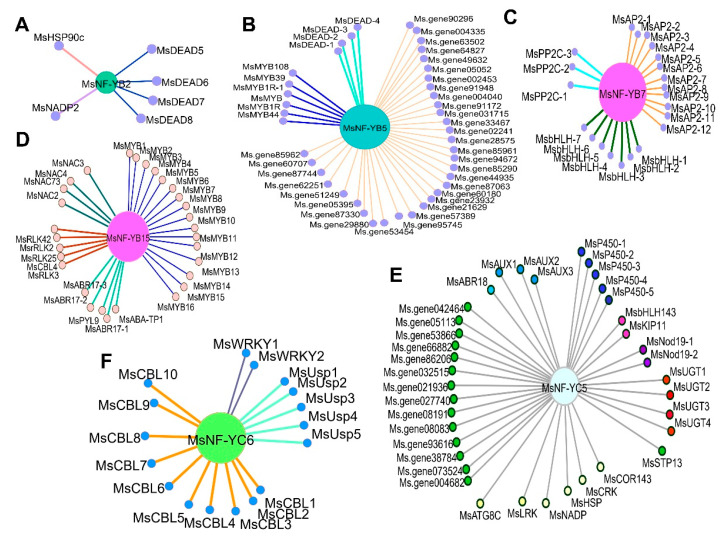
Coexpression network of the six *MsNF-Y* genes under salt stress. (**A**) The network of *MsNF-YB2*, (**B**) *MsNF-YB5*, (**C**) *MsNF-YB7*, (**D**) *MsNF-YB15*, (**E**) *MsNF-YC5*, and (**F**) *MsNF-YC6*.

To further understand the coexpression network of the six *MsNF-Y* genes, the genes that were synergistically expressed under salt stress were identified. The results showed that *MsNF-YB2* was mainly coexpressed with the DEAD genes under salt stress at the early stage (Figure 10A and Table S3). Regarding *MsNF-YB5*, there were 43 coexpressed genes, including 33 protein kinase genes, 6 MYB transcription factors and 4 DEAD genes (Figure 10B and Table S4). In total, 22 coexpressed genes containing 12 AP2 domain genes, 7 bHLH transcription factors, and 3 PP2C genes with *MsNF-YB7* were found (Figure 10C and Table S5). Regarding *MsNF-YB15*, there were 30 coexpressed genes, which were mainly MYB transcription factors (Figure 10D and Table S6). In total, 38 genes were found to be coexpressed with *MsNF-YC5* (Figure 10E and Table S7). Regarding *MsNF-YC6*, 17 genes were coexpressed. Among them, 10 genes encode CBL-interacting serine/threonine-protein kinases, indicating that *MsNF-YC6* may be involved in the Ca^2+^ signaling process under salt stress (Figure 10F and Table S8). These results provide information for a regulatory network analysis of these *MsNF-Y* genes.

### 2.10. RT-qPCR Analysis of MsNF-Y Genes under Salt Conditions

**Figure 11:**
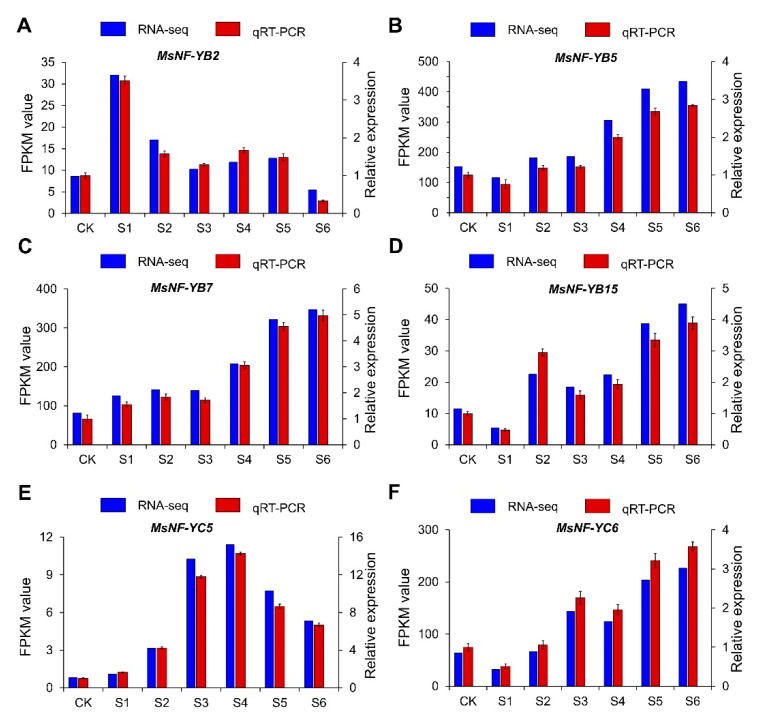
qRT-PCR analysis for genes related salt stress. (**A**) *MsNF-YB2*, (**B**) *MsNF-YB5*, (**C**) *MsNF-YB7*, (**D**) *MsNF-YB15*, (**E**) *MsNF-YC5*, and (**F**) *MsNF-YC6*.

To confirm the salt-induced expression profiles of six *MsNF-Y* genes, a RT-qPCR was further applied. As shown in Figure 11, the expression profiles of the candidate genes were consistent with RNA-Seq results. The expression of *MsNF-YB2* was significantly up-regulated at S1 stage and then decreased gradually. The expressions of *MsNF-YB5*, *MsNF-YB7*, *MsNF-YB15*, and *MsNF-YC6* were increased gradually after salt treatment and reached the highest at S6 stage. The expression of *MsNF-YC5* was comparatively high at S4 and S5 stage. The RT-qPCR results verified the results of RNA-Seq and indicated that these six *MsNF-Y* genes identified by our analyses were important candidate genes and may play roles in response to salt stress in alfalfa.

## 3. Discussion

As a gene family important for plant growth and development and abiotic stress resistance, NF-Y transcription factors have been identified and functionally analyzed genome-wide in many plant species. For example, 36 *NF-Y* gene members (10 *NF-YAs*, 13 *NF-YBs*, and 13 *NF-YCs*) were identified in *Arabidopsis* [25], 78 *NF-Y* gene members were identified in soybean (21 *NF-Yas*, 32 *NF-Ybs*, and 25 *NF-YCs*) [19], 34 *NF-Y* gene members were identified in rice (11 *NF-YAs*, 11 *NF-YBs*, and 12 *NF-YCs*) [26], and 50 *NF-Y* gene members were identified in maize (14 *NF-Yas*, 18 *NF-Ybs*, and 18 *NF-YCs*) [27]. However, because alfalfa is autotetraploid, genome assembly is difficult, and it was not until 2020 that the alfalfa reference genome was assembled successfully [1]. In this study, in total, 60 *MsNF-Y* members (9 *NF-YAs*, 26 *NF-YBs*, and 25 *NF-YCs*) were identified in the alfalfa reference genome, and their gene structures, sequence features, conserved domains, and expression patterns were systematically analyzed, particularly the expression changes of *MsNF-Ys* under different abiotic stresses. These findings provide valuable information for a subsequent functional analysis and molecular regulatory network construction of a single *MsNF-Y* gene.

Many studies provide evidence suggesting that NF-Y transcription factors are widely involved in plant flowering time regulation and seed development. The overexpression of *AtNF-YB1* in *Arabidopsis* resulted in delayed flowering in plants [28], while a phylogenetic analysis found that MsNF-YB1/2/3/4 had high homology with AtNF-YB1 (Figure 6B). Furthermore, through an expression pattern analysis, *MsNF-YB3* was found to be specifically expressed in flowers (Figure 7B); thus, we inferred that *MsNF-YB3* might be an important candidate gene for flowering time regulation in alfalfa. *AtNF-YB9* regulates seed development by integrating light signals with hormonal signals, and the homologous gene *AtNF-YB6* is involved in the morphogenesis of seed embryos by mediating the ABA signaling pathway [28]. In the present study, we found that MsNF-YB20/22/23/25 and AtNF-YB6/9 were clustered in one evolutionary branch (Figure 6B); therefore, the four *MsNF-YB* genes might also be involved in the seed development process in alfalfa.

The NF-Y transcription factor is not only involved in plant growth and development but also plays an important role in plant abiotic stress [4]. The overexpression of *AtNF-YB1* and *ZmNF-YB2* in *Arabidopsis* and maize, respectively, significantly improved drought resistance in the transgenic lines [10,28]. Similarly, the overexpression of soybean *GmNF-YA3* or poplar *PdNF-YB7* in *Arabidopsis* can reduce leaf water loss and improve plant drought resistance [29,30]. The overexpression of *AtNF-YA1* can significantly improve the resistance of *Arabidopsis* to salt stress [28]. *GmNF-YA16*, *GmNF-YB2*, *GmNF-YC13*, and *GmNF-YC14* in soybean can significantly enhance the resistance of plants to drought and salt stress [19,22,29]. In this study, *MsNF-YB14* and *MsNF-YB15* were homologous genes of *GmNF-YB2*, while *MsNF-YC5* and *MsNF-YC6* were homologous genes of *GmNF-YC13* (Figure 6). Their expression was induced by drought and salt stress (Figure 8). Therefore, *MsNF-YB14/15* and *MsNF-YC5*/*6* may also perform the function of regulating alfalfa resistance to drought and salt stress.

NF-Y transcription factors regulate drought and salt stress in plants by mediating multiple signaling and plant hormone pathways. The regulatory pathways mainly include two types. One type mediates the ABA signaling pathway to regulate expression changes in downstream genes. For example, PdNF-YB21 can interact with PdFUS3 to jointly activate the expression of downstream *PdNCED3* genes, thereby promoting the synthesis of ABA and ultimately improving drought tolerance in poplars [31]. In soybean, GmNF-YC14 can interact with GmNF-YB2 and GmNF-YA16 to form a complete, active NF-Y transcriptional complex, and this complex can regulate the ABA signaling pathway mediated by the ABA receptor PYR/PYL to enhance drought resistance and salt tolerance in soybeans [22]. WGCNA, a method commonly used to identify coexpression regulatory networks, has been shown to be highly effective in many studies [32]. In this study, we used WGCNA and found that *MsNF-YB2* was coexpressed with four DEAD genes in the ABA signaling pathway under salt stress, while *MsNF-YB7* was coexpressed with three PP2C genes in the ABA pathway under salt stress (Figure 10). Another regulatory pathway of *NF-Y* genes alters the stress resistance ability of plants by mediating independent ABA pathway genes. After overexpressing *AtNF-YB1* in *Arabidopsis*, although drought resistance was improved in the transgenic lines, there was no significant change in drought resistance genes related to the ABA pathway, suggesting that NF-Y family members exist in a drought resistance pathway independent of the ABA signaling pathway [10,28]. In this study, many genes independent of the ABA pathway were found to be coexpressed with the *MsNF-Y* genes under drought and salt stresses. For example, 6 MYB transcription factors were coexpressed with *MsNF-YB5*, 16 MYB transcription factors were coexpressed with *MsNF-YB15*, and 10 CBL genes were coexpressed with the *MsNF-YC6* under salt stress (Figure 10). These results could greatly enhance our understanding of the molecular regulatory network of *MsNF-Y* genes in the future, especially under abiotic stress.

In summary, we provide genome-wide results of the alfalfa *NF-Y* genes and expression patterns and coexpression regulatory networks in different tissues under different abiotic stresses. The information described here could contribute to further studies investigating alfalfa *NF-Y* gene families, especially in the context of abiotic stress.

## 4. Materials and Methods

### 4.1. Identification of NF-Y Genes in the Alfalfa Genome

The reference genome of Xinjiangdaye was obtained from https://figshare.com/ (accessed on 14 November 2021). The HMMER3.0 program was used to identify the *NF-Y* gene family members in alfalfa based on e < 10^−10^ [33]. The protein sequences and the conserved domains of the NF-Y subfamilies were collected as described in Siefers et al. [25]. The candidate NF-Y gene family members in alfalfa were screened via CD-search (https://www.ncbi.nlm.nih.gov/Structure/cdd/wrpsb.cgi, accessed on 22 December 2021) by default to analyze the presence of the conserved domains. The molecular weight (MW) and isoelectric point (pI) were analyzed by ExPASy (https://web.expasy.org, accessed on 29 December 2021).

### 4.2. Motif Analysis and Sequence Alignments

A motif analysis was conducted with the online program MEME (https://meme-suite.org/, accessed on 10 January 2022) by using the protein sequences of these *NF-Y* gene family members [34]. The gene structure was obtained by using TBtools software [35]. DNAMAN software was used to conduct the multiple sequence alignments.

### 4.3. Phylogenetic Tree Construction, Genome Localization and Gene Duplication

The *NF-Y* gene family sequences from *Arabidopsis*, rice, soybean, and Tartary buckwheat were collected from previous studies [21,24,25,26]. The neighbor-joining method with 1000 replicated bootstrap values was used to conduct a phylogenetic analysis by using MEGA 7.0 software based on the protein sequences. A duplication event analysis, including the parameters Ka and Ks, and a genome localization analysis were conducted by TBtools [35].

### 4.4. cis-Element Analysis

The 2 kb sequences upstream of ATG, which is the translation start codon of all *NF-Y* gene family members, were downloaded from the Xinjiangdaye reference genome by using TBtools [35]. Then, the *cis* elements of these sequences were analyzed using the online program PlantCARE, http://bioinformatics.psb.ugent.be/webtools/plantcare/html/ (accessed on 8 February 2022) [36].

### 4.5. Transcription Data Analysis

Transcription data in different tissues (ES1, elongated stem; flower; leaf; nodule; PES, pre-elongated stem; root) were downloaded from the public NCBI database. The reads can be downloaded from the NCBI short read archive database under accession SRP055547 [37]. The RNA-Seq data of alfalfa under different abiotic stresses were available in the NCBI short read archive database (cold, accession SRR7091780-SRR7091794; drought, salt, and ABA, accession SRR7160313-SRR7160357) [38]. Here, the alfalfa accession Zhongmu No. 1 was planted in a plant growth chamber that was maintained at 16 h/8 h (light/dark) and 22 °C for 10 days. For cold stress, the seedlings were maintained at 4 °C for 0 h (CK), 2 h (C1), 6 h (C2), 24 (C3), and 48 h (C4) [38]. For the ABA treatment, 10 µΜ ABA in 1/2 MS nutrient solution were used. The root tips were collected at 0 h (CK), 1 h (ABA_1), 3 h (ABA_2), and 12 h (ABA_3) after the ABA treatment [38]. For drought stress, 400 mM mannitol were used in 1/2 MS nutrient solution. The root tips were collected at 0 h (CK), 1 h (M1), 3 h (M2), 6 h (M3), 12 h (M4), and 24 h (M5) [38]. For salt stress, 250 mM NaCl were used in a 1/2 MS nutrient solution. The root tips were collected at 0 h (CK), 0.5 h (S1), 1 h (S2), 3 h (S3), 6 h (S4), 12 h (S5), and 24 h (S6) [38]. The clean reads were mapped to the “Xinjiangdaye” genome by using TopHat2 software [39]. The gene expression level was estimated by using the FPKM value (fragments per kilobase of transcript per million reads mapped). The DEGs were obtained by using DESeq software based on p_adj_ < 0.05 and |log_2_FC| ≥ 1 [40].

### 4.6. Weighted Gene Coexpression Network Construction

We analyzed and constructed the weighted gene coexpression network (WGCNA) of alfalfa under salt stress using TBtools with the default parameters [32,35]. First, using transcriptome data (salt stress) collected at seven different time points at 0 h (CK), 0.5 h (S1), 1 h (S2), 3 h (S3), 6 h (S4), 12 h (S5), and 24 h (S6), all expressed genes were filtered based on FPKM values (more than 1) for the subsequent analysis. Then, the correlation between different modules and the salt stress treatment time was obtained using a cluster analysis and correlation analysis. Finally, coexpression network drawing was performed using Cytoscape software [41].

### 4.7. Expression Analysis by qRT-PCR

To further verify the expression of candidate genes under salt stress, we collected the root tips of Zhongmu No.1 under NaCl treatment at 0 h (CK), 0.5 h (S1), 1 h (S2), 3 h (S3), 6 h (S4), 12 h (S5), and 24 h (S6) according to a previous study [38]. All the samples were three biological repetitions. Total RNAs were extracted using an RNAprep Pure Plant Kit (Tiangen Biotech) and cDNAs were prepared using Tran-Script-Uni One-Step gDNA Removal and cDNA Synthesis SuperMix (TRAN). Then, we performed qPCR using a SYBR Green RT-PCR Kit (Takara). The primers were designed by Primer Premier 5 software, and the primer sequences were presented in Table S9. The qRT-PCR program was: 95 °C for 30 s; 40 cycles of 95 °C for 5 s; 58 °C for 34 s; and 95 °C for 15 s. *MsActin* was used to as the internal reference gene for data normalization analysis. For each analysis, three technical replicates from three biological replicates were conducted. Ultimately, we calculated the relative expression of *MsNF-Ys* by the 2^−∆∆Ct^ method.

## 5. Conclusions

In our study, in total, 60 *MsNF-Y* genes, including 9 *MsNF-YAs*, 26 *MsNF-YBs*, and 25 *MsNF-YCs*, were identified in the alfalfa reference genome, and conserved domain, gene structure, genomic location, *cis* element, and expression pattern analyses were conducted. The expression analysis showed that 48 *MsNF-Y* genes were expressed in all tissues at different levels, showing that these *MsNF-Y* genes play an important role in alfalfa growth and development. In addition, we analyzed the expression of *MsNF-Y* genes under different abiotic stresses (cold, drought, and salt). The results indicated that a few *MsNF-Y* genes were involved in these abiotic stresses. Finally, the co-expression networks under salt stress of *MsNF-YB2*, *MsNF-YB5*, *MsNF-YB7*, *MsNF-YB15*, *MsNF-YC5*, and *MsNF-YC6* were constructed by using a WGCNA. In future studies, these candidate genes should be over-expressed or gene knocked out in alfalfa to verify their biological functions and the yeast two hybrid system or Chip-seq should be used to identify the interaction network for these candidate genes. Overall, our results could provide valuable information for further elucidating the biological functions of *MsNF-Ys* and improving salt tolerance and other abiotic stress resistance in alfalfa.

## Figures and Tables

**Table 1:** Information of the *MsNF-Y* genes in alfalfa.

Name	Gene ID	CDs	Length (AA)	Chromosome Localization	Mw (kDa)	pI	Homologs in *Arabidopsis*
*MsNF-YA1*	MS.gene84539	1002	333	chr3.1:46159735:46162043	36.08	5.68	*AtNF-YA1/9*
*MsNF-YA2*	MS.gene04098	990	329	chr3.2:52938927:52941216	35.83	5.81	*AtNF-YA1/9*
*MsNF-YA3*	MS.gene08079	990	329	chr3.3:49398206:49400492	35.79	5.81	*AtNF-YA1/9*
*MsNF-YA4*	MS.gene04396	990	329	chr3.4:57338770:57341055	35.77	5.81	*AtNF-YA1/9*
*MsNF-YA5*	MS.gene007115	1005	334	chr7.1:6286954:6289818	36.28	9.48	*AtNF-YA10*
*MsNF-YA6*	MS.gene23087	1005	334	chr7.2:7507261:7510125	36.31	9.48	*AtNF-YA10*
*MsNF-YA7*	MS.gene26493	1005	334	chr7.3:7757499:7760361	36.28	9.48	*AtNF-YA10*
*MsNF-YA8*	MS.gene011285	939	312	chr8.2:75039696:75042162	33.23	7.70	*AtNF-YA6*
*MsNF-YA9*	MS.gene53329	957	318	chr8.4:73707595:73710074	35.03	8.48	*AtNF-YA6*
*MsNF-YB1*	MS.gene004698	450	149	chr1.1:54900382:54901482	16.34	5.32	*AtNF-YB1/8/10*
*MsNF-YB2*	MS.gene32082	444	147	chr1.2:55567170:55568359	16.09	5.54	*AtNF-YB8/10*
*MsNF-YB3*	MS.gene036253	531	176	chr1.3:52425710:52429054	19.47	5.56	*AtNF-YB1/8/10*
*MsNF-YB4*	MS.gene40015	450	149	chr1.4:59578396:59579496	16.34	5.32	*AtNF-YB1/8/10*
*MsNF-YB5*	MS.gene045804	411	136	chr2.1:36197735:36200699	15.28	4.61	*AtNF-YB12/13*
*MsNF-YB6*	MS.gene048249	513	170	chr2.3:62568258:62569485	18.58	5.48	*AtNF-YB2/3*
*MsNF-YB7*	MS.gene83228	471	156	chr2.4:34709719:34712823	17.42	4.63	*AtNF-YB12/13*
*MsNF-YB8*	MS.gene02911	489	162	chr2.4:62033643:62034131	17.67	5.78	*AtNF-YB3*
*MsNF-YB9*	MS.gene94950	570	189	chr3.1:44574260:44574829	20.12	6.21	*AtNF-YB2/3*
*MsNF-YB10*	MS.gene05647	570	189	chr3.2:51228991:51229560	20.12	6.21	*AtNF-YB2/3*
*MsNF-YB11*	MS.gene04197	570	189	chr3.3:48302965:48303534	20.06	6.21	*AtNF-YB2/3*
*MsNF-YB12*	MS.gene04298	570	189	chr3.4:55907374:55907943	20.12	6.21	*AtNF-YB2/3*
*MsNF-YB13*	MS.gene37951	558	185	chr5.1:76486331:76486888	20.21	5.87	*AtNF-YB2/3*
*MsNF-YB14*	MS.gene81327	558	185	chr5.2:80485723:80486280	20.32	6.23	*AtNF-YB2/3*
*MsNF-YB15*	MS.gene020793	558	185	chr5.3:77328038:77328595	20.20	5.75	*AtNF-YB2/3*
*MsNF-YB16*	MS.gene22469	510	169	chr7.1:8232375:8233608	18.62	8.49	*AtNF-YB1/8/10*
*MsNF-YB17*	MS.gene20033	510	169	chr7.2:11459416:11460648	18.62	8.49	*AtNF-YB1/8/10*
*MsNF-YB18*	MS.gene007467	510	169	chr7.2:11511734:11512962	18.62	8.49	*AtNF-YB1/8/10*
*MsNF-YB19*	MS.gene09802	510	169	chr7.3:12232958:12234190	18.62	8.49	*AtNF-YB1/8/10*
*MsNF-YB20*	MS.gene012740	714	237	chr8.1:730789:733261	26.28	6.26	*AtNF-YB6*
*MsNF-YB21*	MS.gene044533	462	153	chr8.1:14534380:14537789	17.15	4.72	*AtNF-YB12/13*
*MsNF-YB22*	MS.gene95097	708	235	chr8.2:735520:737984	26.14	6.26	*AtNF-YB6*
*MsNF-YB23*	MS.gene042085	708	235	chr8.3:558353:560819	26.14	6.26	*AtNF-YB6*
*MsNF-YB24*	MS.gene038788	462	153	chr8.3:14583864:14587257	17.15	4.72	*AtNF-YB12/13*
*MsNF-YB25*	MS.gene43428	708	235	chr8.4:784437:786904	26.14	6.26	*AtNF-YB6*
*MsNF-YB26*	MS.gene033001	462	153	chr8.4:17356790:17360381	17.15	4.72	*AtNF-YB12/13*
*MsNF-YC1*	MS.gene96763	768	255	chr1.1:61200434:61201201	28.41	5.62	*AtNF-YC1/2/3/9*
*MsNF-YC2*	MS.gene055556	768	255	chr1.2:60238503:60239270	28.40	5.62	*AtNF-YC1/2/3/9*
*MsNF-YC3*	MS.gene44078	768	255	chr1.3:58221159:58221926	28.40	5.62	*AtNF-YC1/2/3/9*
*MsNF-YC4*	MS.gene005213	768	255	chr1.4:66923142:66923909	28.40	5.62	*AtNF-YC1/2/3/9*
*MsNF-YC5*	MS.gene073979	798	265	chr2.1:30297070:30297867	29.76	5.79	*AtNF-YC1/2/3/4/9*
*MsNF-YC6*	MS.gene039020	804	267	chr2.2:24489042:24489845	30.13	5.97	*AtNF-YC1/2/3/4/9*
*MsNF-YC7*	MS.gene77621	798	265	chr2.3:30013127:30013924	29.85	5.97	*AtNF-YC1/2/3/4/9*
*MsNF-YC8*	MS.gene92165	798	265	chr2.4:27659056:27659853	29.82	5.79	*AtNF-YC1/2/3/4/9*
*MsNF-YC9*	MS.gene49550	891	296	chr3.1:8392449:8394316	32.70	5.66	*AtNF-YC11*
*MsNF-YC10*	MS.gene039544	1140	379	chr3.1:8402787:8408425	41.59	5.75	*AtNF-YC11*
*MsNF-YC11*	MS.gene06223	678	225	chr3.1:79056763:79057440	25.17	5.65	*AtNF-YC1/4*
*MsNF-YC12*	MS.gene97173	1140	379	chr3.2:6048908:6054534	41.60	5.75	*AtNF-YC11*
*MsNF-YC13*	MS.gene055499	570	189	chr3.2:82525648:82526217	20.97	5.71	*AtNF-YC1/4*
*MsNF-YC14*	MS.gene88253	1203	400	chr3.3:7831439:7839020	43.94	8.95	*AtNF-YC11*
*MsNF-YC15*	MS.gene013147	483	160	chr3.3:81706695:81707177	17.32	4.96	*AtNF-YC1/4*
*MsNF-YC16*	MS.gene046309	1140	379	chr3.4:8318736:8325208	41.60	5.75	*AtNF-YC11*
*MsNF-YC17*	MS.gene050860	783	260	chr7.1:2796742:2797524	29.04	6.03	*AtNF-YC1/2/3/4/9*
*MsNF-YC18*	MS.gene29325	783	260	chr7.2:3652860:3653642	29.04	6.03	*AtNF-YC1/2/3/4/9*
*MsNF-YC19*	MS.gene67561	783	260	chr7.3:3320464:3321246	29.04	6.03	*AtNF-YC1/2/3/4/9*
*MsNF-YC20*	MS.gene050859	723	240	chr7.3:3331958:3332680	26.95	6.04	*AtNF-YC1/2/3/4/9*
*MsNF-YC21*	MS.gene072458	783	260	chr7.3:3363915:3364697	29.04	6.03	*AtNF-YC1/2/3/4/9*
*MsNF-YC22*	MS.gene072460	783	260	chr7.3:3435457:3436239	29.04	6.03	*AtNF-YC1/2/3/4/9*
*MsNF-YC23*	MS.gene063839	918	305	chr8.1:62879212:62882462	33.26	5.08	*AtNF-YC11*
*MsNF-YC24*	MS.gene88394	918	305	chr8.3:56793662:56796860	33.37	5.13	*AtNF-YC11*
*MsNF-YC25*	MS.gene78092	918	305	chr8.4:58034595:58037795	33.39	5.02	*AtNF-YC11*

## Data Availability

The data that support the findings of this study are available from the corresponding author upon reasonable request.

## Supplementary Materials

The following supporting information can be downloaded at https://www.mdpi.com/article/10.3390/ijms23126426/s1.
